# Micro- and nanoplastic toxicity in humans: Exposure pathways, cellular effects, and mitigation strategies

**DOI:** 10.1016/j.toxrep.2025.102043

**Published:** 2025-05-10

**Authors:** Faezeh Jahedi, Neamatollah Jaafarzadeh Haghighi Fard

**Affiliations:** a Department of Environmental Health Engineering, School of Health, Ahvaz Jundishapur University of Medical Sciences, Ahvaz, Iran; b Student Research Committee, Ahvaz Jundishapur University of Medical Sciences, Ahvaz, Iran; c Environmental Technologies Research Center, Medical Basic Sciences Research Institute, Ahvaz Jundishapur University of Medical Sciences, Ahvaz, Iran

**Keywords:** Microplastics, Nanoplastics, Human exposure, Toxicity, Inflammation, Metabolic disorders, Neurotoxicity, Detoxification strategies

## Abstract

Microplastics and nanoplastics (MNPs) are emerging environmental contaminants with increasing scientific evidence suggesting their potential risks to human health. The present review systematically explores the pathways through which these particles enter the human body, the cellular and molecular mechanisms of their toxicity, and current strategies to mitigate their effects. A structured literature review was conducted following PRISMA guidelines, focusing on studies published between 2019 and 2024 across major scientific databases. MNPs primarily enter the human system via ingestion, inhalation, and dermal exposure. Once internalized, they can accumulate in various organs and trigger oxidative stress, inflammation, apoptosis, and genotoxic effects. These toxic responses have been linked to chronic conditions such as metabolic disorders (e.g., diabetes, obesity), immune dysfunction, and neurodegenerative diseases. Furthermore, this review highlights emerging attenuation strategies, including advanced filtration technologies, bioremediation approaches, and bioactive compounds such as melatonin, astaxanthin, and probiotics. By identifying exposure pathways, toxic effects, and current research gaps, this review provides a comprehensive foundation for developing effective interventions to reduce MNP-related health risks and inform future toxicological studies.

## 1 Introduction

Since the 1950s, global plastic production has risen dramatically, reaching approximately 390.7 million metric tons in 2021 and projected to exceed 1100 million metric tons by 2050 [1], [2], [3], [4], [5]. This surge has led to the persistent accumulation of plastic waste in natural environments, where degradation processes result in the formation of microplastics)particles smaller than 5 mm) and nanoplastics (less than 100 nm)[6]. These small plastic particles are now ubiquitous in terrestrial and aquatic ecosystems and have raised increasing concern for their entry into the human body and associated health effects[7], [8]. Recent studies have documented the presence of MNPs in drinking water (tap and bottled), seafood, salt, air, and even human tissues such as the lungs, colon, placenta, and blood. For example, a global study revealed that bottled water can contain up to 10,000 microplastic particles per liter[9]. Similarly, indoor air concentrations of microplastic fibers may reach hundreds to thousands of particles per cubic meter, particularly in urban environments[10], [11], [12], [13]. Several review articles have explored the environmental fate and ecotoxicology of microplastics, primarily focusing on marine organisms or environmental compartments. However, relatively few reviews have comprehensively addressed the toxicological mechanisms of MNPs in humans, nor have they sufficiently explored strategies to mitigate their toxicity. MNPs have been found in the human colon[14], [15], placenta[16], lung tissue [17], [18], [19], [20], and stool [21]. This discovery underscores the serious and emerging challenges that MNPs pose to both environmental and public health[22].

Most existing reviews focus on environmental contamination or merely summarize exposure sources. Few have integrated emerging molecular evidence or discussed therapeutic interventions.

This review aims to address these gaps by providing a structured synthesis of (i) the primary human exposure routes to MNPs, (ii) evidence of their accumulation in human tissues, (iii) known and proposed toxicological effects on cellular and systemic levels, and (iv) current and emerging strategies to reduce their impact. By consolidating this knowledge, we aim to support future toxicological studies and inform the development of protective public health measures.

## 2 Materials and methods

This review was conducted following the Preferred Reporting Items for Systematic Reviews and Meta-Analyses (PRISMA) guidelines. A comprehensive literature search was carried out to identify peer-reviewed studies on microplastic and nanoplastic exposure and toxicity in humans.

### 2.1 Search strategy

Databases searched included PubMed, Scopus, and Web of Science. The search covered publications from January 1, 2019, to January 31, 2024, using combinations of the following keywords:

• “microplastics” OR “nanoplastics”
• AND “toxicity” OR “health effects”
• AND “human exposure” OR “ingestion” OR “inhalation” OR “dermal”
• AND “oxidative stress” OR “inflammation” OR “apoptosis” OR “mitigation”

Boolean operators (AND, OR) were applied to refine results. Only English-language articles published in peer-reviewed journals were considered.

### 2.2 Inclusion criteria

• Original research articles (in vitro or in vivo) involving human or mammalian models
• Studies reporting toxicological effects or biological accumulation of MNPs
• Studies describing attenuation strategies or exposure mitigation

### 2.3 Exclusion criteria

• Review articles, editorials, and conference abstracts
• Studies focusing solely on marine organisms or non-human environmental toxicology
• Articles without accessible full text

### 2.4 Screening and selection

Title and abstract screening was independently performed by two reviewers, followed by full-text review. Discrepancies were resolved through discussion and consensus.

**Fig. 1:**
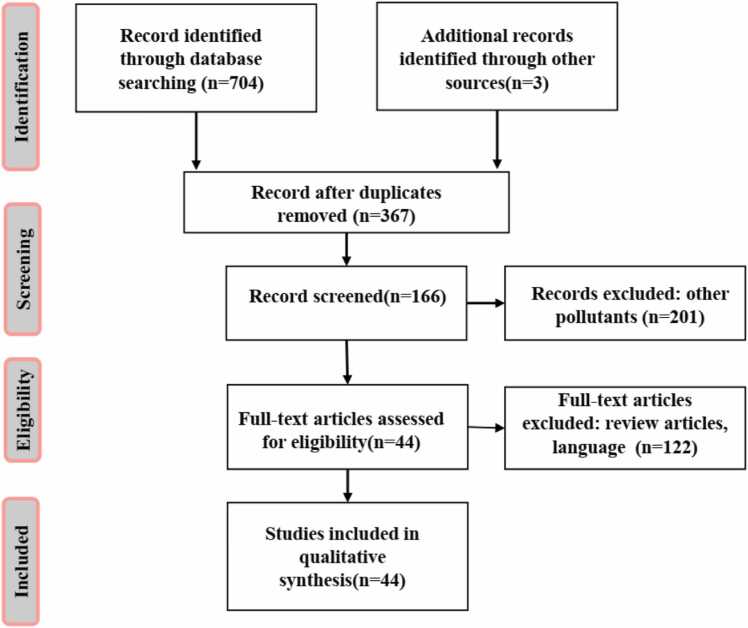
Summary of protocol for this literature review.

Disagreements were resolved by discussion and consensus. A PRISMA flow diagram (Fig. 1) illustrates the selection process.

## 3 Results and discussion

### 3.1 MNPs exposure routes

Human exposure to microplastics and nanoplastics (MNPs) can occur through various routes, including ingestion, inhalation, dermal contact, injection, and implantation.

#### 3.1.1 Ingestion

Ingestion remains the most documented exposure route, primarily through contaminated food and water[23]. Extensive research has documented the presence of MNPs in a wide range of foods, including bivalves[24], [25], [26], crustaceans and commercial fish[27], [28], sugar [29], salt [30], honey [31], tea[32] and bottled water [33]. Nanoplastics (<100 nm) are particularly concerning due to their ability to cross the intestinal barrier and enter systemic circulation. Hydrophobic polymers like polystyrene (PS) and polyethylene (PE) tend to interact more with lipophilic cell membranes, potentially enhancing uptake. Furthermore, surface functionalization (e.g., oxidized vs. unmodified plastics) can affect their bioavailability and internalization in the gastrointestinal tract.

#### 3.1.2 Inhalation

Inhalation represents another major route of exposure. MNPs are prevalent in both indoor and outdoor air, originating from sources such as synthetic clothing and textiles[34], shedding from building materials, abrasions from plastic products [35], waste incineration, and landfilling [36]. Numerous studies have detected MNPs in human samples, including lungs, sputum, and lavage fluid, underscoring inhalation as a significant pathway for MNP exposure. Synthetic textile fibers (polyester, acrylic), plastic dust, and urban air particles contribute to microplastic presence in the air. Nanoplastics, due to their small size and aerodynamic diameter, can reach the alveoli and potentially enter the bloodstream. The shape and density of particles (e.g., fibers vs. spheres) also influence deposition efficiency in the respiratory tract. Polymers such as polyvinyl chloride (PVC), commonly released during industrial combustion, are known to produce respirable particles that may induce oxidative stress and inflammatory responses in lung tissue.[17], [37], [38].

#### 3.1.3 Dermal and transdermal absorption

Dermal contact is also a notable route of exposure to MNPs. Humans can absorb MNPs through the skin via the use of cosmetics, body washes, hand and face masks[4], [39], topical medications, surgical and prosthetic devices, injections, implants, sunscreens [40], and toothpaste [41]. Research has shown that dermal exposure to MNPs can induce oxidative stress in human epithelial cells. Additionally, plastic products used in surgical procedures and body prosthetics can cause local inflammation and other adverse reactions[42]. Recent studies have highlighted that the mechanical wear of medical devices implanted in the human body, such as polyethylene joint spacers in shoulder, knee, or hip replacements, dental implants and caps, and cosmetic implants, can generate MNP particles. These particles can subsequently be transferred into the body, further contributing to the overall exposure to MNPs. This route of exposure is particularly concerning due to the direct and prolonged contact with internal tissues[43], [44], [45], [46].

### 3.2 Accumulation of MNPs in human organs

**Fig. 2:**
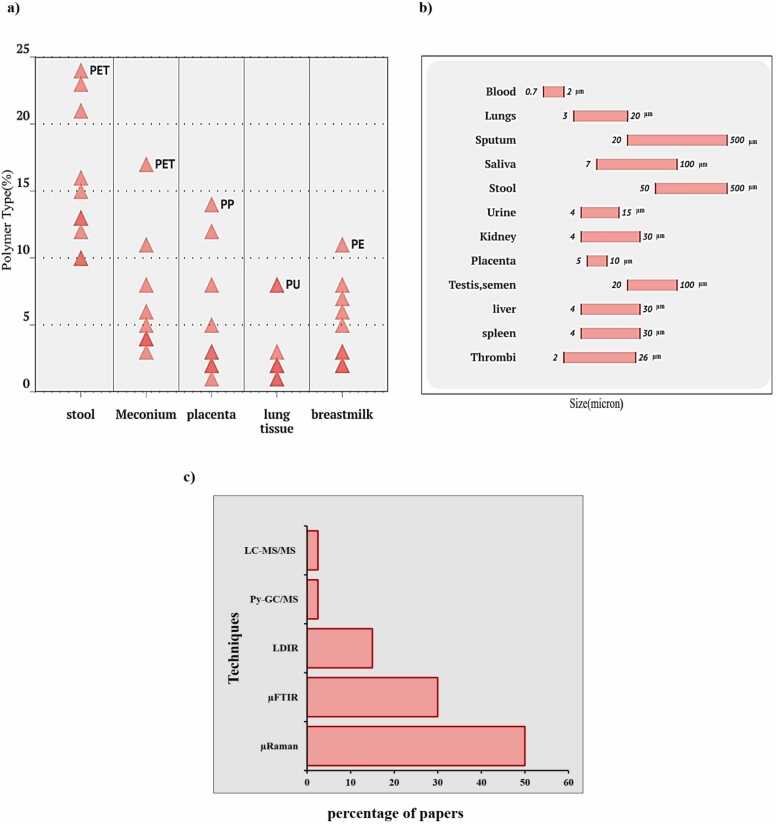
a) The predominant polymer identified in various human biological samples analyzed in the studies included in this review. b) The size of MNPs identified in different human biological samples analyzed in the studies included in this review. c) The technique used in the identification of MNPs in the articles included in this review.

Many studies have confirmed the presence of MNPs in the human body Including sputum, saliva, lavage, lung tissue, liver, kidney, placenta, intestine, and feces, etc. which are summarized in Table1. In a study, they investigated the presence of MNPs in the blood, and its amount was on average 1.6 mg/mL of the total measurable concentration of MNPs in the blood. In this study, the most common ones were polyethylene terephthalate, polyethylene, styrene polymers and, poly (methyl acrylate)[47]. Jenner and colleagues showed that MNPs were detected with a rate of 1.42 + 1.50[37]. In a study conducted by Amato Lourenco et al., 37 types of MNPs were identified in 20 lung tissue samples[17]. In a study conducted by Huang et al., the sputum samples of 22 respiratory patients with hospital admission were examined along with the completion of a checklist of personal information and lifestyle; The results of this study show the presence of MNPs in all the samples with 21 different types of chemical composition with a size of 20–500 μm. Most of the MNPs in the samples were determined to be polyurethane[38]. In Table 1, a summary of the studies related to the identification of MNPs in the human body is given, as it is very clear, for example, MNPs of stool samples consisting of PS, PET, and PP and the most type of PET are reported (Fig. 2a). This scatter plot shows the percentage of different polymer types found in various human samples. The x-axis categories include stool, meconium (newborn’s first stool), placenta, lung, and breast milk. The y-axis represents the percentage of polymer types, ranging from 0 % to 25 %. The red triangular data points indicate the presence of different polymers in each category. About the placenta, all studies identified MNPs from PP, and some other studies reported PA, PET, PU, and PE. In studies related to meconium, PET was the most predominant type of MNP identified. Studies focused on lung tissue samples, polyurethane, and PE has been reported as the most dominant type of polymer in all breast milk studies. Fig2b consists of three columns: body samples, particle size, and particle concentration. The body samples include blood, sputum, lungs, saliva, urine, stool, placenta, kidney, testis/semen, liver, spleen, and thrombi. The particle size lists various sizes of micro- and nanoplastic particles, such as 2 µm, 20 µm, etc. The particle concentration shows the concentration of particles, ranging from 10 µm to 500 µm. These charts indicate that micro- and nanoplastic particles have been found in various human body samples, with different sizes and concentrations depending on the sample type. It was also found that in studies to identify MNPs in human biological samples, about 50 % of µ-Raman spectroscopic analysis and then m-Fourier transform infrared spectroscopy (µ-FT-IR) analysis and laser direct infrared (LDIR) respectively. pyrolysis gas chromatography-mass spectrometry (Py-GCMS) and liquid chromatography-tandem mass spectrometry (LC-MS/MS) (Fig. 2C)

**Table 1:** A summary of the studies in this review that identified MNPs in the biological sample of the human body.

Human tissue	Sampling	Concentration	Composition	Size	Detection	Ref
Blood	22 healthy, non-fasting adult volunteers	1.6 mg/mL	PMMA, PP, PS, PE,PET	< 700 nm	pyrolysis- gas chromatography/mass spectrometry)Py-GC/MS(	[48]
blood, cerebrospinal fluid, effusions and cyst fluids	104 patients aged 24–96 years with an average age of 56 years	702 microparticles/l	PP, PS, PTFE, PVB, PA, LDPE, PEAA, PSAN,PVA	2.15–103.27 µm	Raman	[49]
Lungs	13 lung tissue samples	1.42 ± 1.50 MP/g of tissue	PAN,PE, PES,PET,PMMA,PP; PS,PTFE, PUR,SEBS,TPE	<3 µm	µFTIR	[37]
Lungs	20 Pulmonary tissue samples from a left lung non-smokers dead individual	mean weight of 3.28 g tissue samples	PP, Cotton, PE, Cellulose acetate, PVC, PS, PA	8.12–16.8 µm	Raman	[17]
Lungs	100 human lung tissues	65 microfibers	-	> 20 μm	μ-FTIR	[50]
lavage fluid	44 adult patients, undergoing a bronchoscopy	9.18 ± 2.45 items/100 mL	Rayon, PS, cellulose, cotton	1.73 ± 0.15 mm	µ-FTIR	[51]
Sputum	22 patients suffering from respiratory diseases	39.5 particles/10 mL (18.75–91.75 particles/10 mL)	Acrylates, AB, alkyd varnish, EVA, PBD, PS, PE, PA, PMMA, PP, PSO, PSU, PU, PVC, rubber, silicone,PC, PTFE	20–500 µm	FTIR	[38]
Saliva, Head Hairs, Face skin, Hand skin	8000 samples from 2000 participants	16,000 particles/8000 samples, (>7000, or, on average, >3.5 MPLs per individual per day	PE, PTFE, PP,PS, PVC	< 100 µm; 100 < L < 250 µm; 250 < L< 500 µm; L > 500 µm)	µ-Raman	[52]
Stool	8 (3 men and 5 women)	20 particles per 10 g	PP, PET, PS, PE, PA, PVC, POM, PC,PU	50–500 µm	FTIR	[21]
Stool	26 male students aged 18–25 years	2200–82,000 ng/g 37–2100 ng/g	PET,PC	-	HPLC-MS/MS	[53]
Stool	50 healthy participants (30men−20women)and 52 IBD participants(31men −21women)	41.8 items/g in patients with inflammatory bowel disease; 28.0 items/g in healthy people	PET, PA, PP, PE, PVC, PS, PC, POM, PTFE, EVA, PMMA, PBT, AS, PES, TPU	1.7 − 393.8 µm	Raman	[54]
Stool, Placentas, meconium, breast milk	18 mothers-infant pairs (18 Placentas, 12 meconium, 7 breast milk samples, 12 stool)	Placentas:18.0 particles/g meconium:54.1 particles/g breast milk: 20.2 particles/g stool: 26.6 particles/g	placenta, meconium and infant feces: PA, breast milk: PU	20–50 μm	Agilent 8700 laser infrared imaging spectrometer (LDIR)	[55]
Meconium	37 newborns	-	-	50 μm and 200 μm	µ-FTIR	[56]
Urine	6pepolr(3men and3 women)	1–3 item/l	PVA, PVC, PP, PE	4–15 µm size)	Raman	[57]
Kidney tissue Urine	10 urines and 10 kidney tissue	Kidney tissue:4.34.11/ Sample Urine: 2.31.56/sample	silicone, graphite, sphere, nylon, PE		Raman	[58]
Placenta	6 human placentas from women with physiological pregnancies	12 fragments in a 23-gram sample out of the total ∼600 g of Placenta	PE	5–10 µm	Raman	[59]
Placenta	43 Placentas	2–38 particles per placenta	PE-PS	< 5 mm	-	[60]
Placenta	17 placentas	2.70 ± 2.65 particles/g	PVC, PP, and PBS	< 200 μm	LD-IR	[61]
Testis& semen	6 testis and 30 semen samples	0.23 ± 0.45 particles/mL in semen and 11.60 ± 15.52 particles/g in testis.	PS-PE-PVC	20–100 µm	Py-GC/MS and LD-IR	[62]
Colon	11 adults men	28.1 ± 15.4 MP/g tissue	PC, PA, PP	-	FTIR	[14]
Semen	10 semen samples	six out of ten	PP, PE, PET,PS, PVC, PC, POM and acrylic	2–6 μm	Raman	[63]
Thrombi	26 thrombi	5 MPs/thrombi (1–15 MPs/thrombi)		2.1–26.0 μm	Raman	[64]
Liver	11sample: 6 patients with liver cirrhosis 5 individuals without underlying liver disease	3.2 MP/g tissue	PS, PVC, PET, PMMA, POM, PP	4–30 µm	µRaman	[65]
Kidney	3samples	0MP	PS, PVC, PET, PMMA, POM, PP	4–30 µm	µRaman	[65]
Spleen	3samples	0.9 MP/g	PS, PVC, PET, PMMA, POM, PP	4–30 µm	µRaman	[65]

### 3.3 Toxicity of MNPs

The toxic effects of microplastics and nanoplastics (MNPs) on human health are mediated by several mechanisms including oxidative stress, inflammation, apoptosis, immune dysregulation, and even genotoxicity. These outcomes depend heavily on particle size, shape, surface chemistry, and polymer type, as well as the exposure route and experimental model used (in vitro vs. in vivo).

#### 3.3.1 In vitro toxicity studies

Cell culture models have been widely used to explore MNP-induced cellular responses. In vitro studies using human epithelial, hepatic, neuronal, and immune cell lines have consistently demonstrated that smaller particles (particularly <100 nm) induce greater oxidative stress and mitochondrial dysfunction compared to larger microplastics. Polystyrene nanoplastics (PS-NPs), for example, have been shown to increase ROS production, reduce cell viability, and promote pro-inflammatory cytokine release in human intestinal and bronchial epithelial cells.

Surface charge and functional groups of MNPs influence their cellular uptake and localization. Positively charged particles are more likely to interact with negatively charged cell membranes, promoting internalization and endosomal escape. Furthermore, polymer type affects intracellular responses—PVC and PS particles generally show higher cytotoxicity than PET or PE in comparable doses, likely due to their associated additives and higher reactivity.

**Fig. 3:**
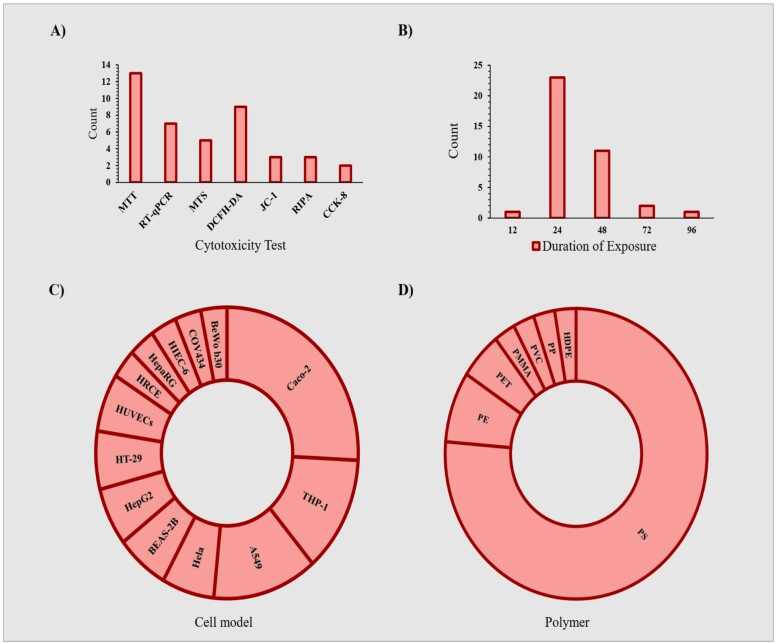
Comparison of (a) cytotoxicity tests, (b) exposure durations(c) cell models (d) polymer types across studies in Table 2.

By comparing the studies in this review (Table 2), which were conducted to evaluate the effects of MNP toxicity on human cells, we find that the number of studies focusing on respiratory and liver intestinal cells is greater than those on other cell types (Fig. 7). The most commonly used cell models in these studies are Caco-2, THP-1, and A549 cells (Fig. 3C). The largest number of cells exposed to MNPs were intestinal, respiratory, and liver cells (Fig. 8), with polystyrene being the most frequently used type of MNP (Fig. 3D). The size of the exposed polystyrene particles ranged from as small as 0.025 µm in placental cells to as large as 10 µm in lung cells (Fig. 6).

**Table 2:** A summary of the studies in this review about the toxicity of MNPs in human cells.

System	Cell	MNPs	Size	Shape	Concentration	Duration	Tests	Result	Ref
Placenta	In vitro, Human Umbilical Vein Epithelial cells (HUVECs)	PS	100–500 nm	sphere	0–100 μg/mL	24 hand 48 h	MTT, Lactate dehydrogenase (LDH), BCA protein assay kit, mCherry-GFP-LC3, SYBR Premix Ex Taq™ II Kit, DCFH-DA	Cell membrane distruction, autophagic flux obstruction, autophagosome formation	[66]
	human umbilical vein endothelial cells HUVECs	PS	0.5, 1, and 5 μm	-	0–100 μg/mL	48 and 72 h	MTT, Angiogenesis Assay kit (ECM625), DCFH-DA, RIPA, BCA,	Suppression of angiogenic signaling pathways and inhibition of wound healing. Decreased viability, increased cell death	[67]
	BeWo b30 cells	PS- HDPE	< 50 μm		100 μg/mL	24 h	RT-qPCR, Lactate dehydrogenase (LDH), and CellTiter-Glo for viability assay	Plasma membrane damage	[68]
	Human placental perfusion model	Carboxylate modified polystyrene particles (50 and 300 nm)	50–300 nm		1–100 μg/mL		MTS	Transfer of polystyrene particles from mother to fetus Polystyrene particles were accumulated in the syncytiotrophoblast of the placenta tissue.	[69]
Granulosa cells	COV434 cells	PS	50 nm		50,100, 150 and 200 µg/mL	24 h	JC−1, DCFH-DA, RIPA	Decreased cell viability and mitochondrial membrane potential, increasing oxidative and apoptotic stress and stopping the next cell cycle	[70]
Intestinal	Human intestinal epithelial cells (Caco−2)	PS-NPLs, PS-COOH, PS-NH2	100 nm	-	30, 60, 120, 240, 480 μg/mL	24 h, 48 h, or 96 h	LDH test, BCA,	hematological system damage	[71]
	Human intestinal epithelial cells (Caco−2)	PS	50 nm		100 μg/mL	24 h	*RT–PCR* , *DCFH-DA*	structural changes in the nucleus and in genotoxicity biomarkers	[72]
	In vitro, Gastric adenocarcinoma (AGS) cells	PS	44 and 100 nm		0–10 μg/100 mL	24 h	MTT, RNA extraction, reverse transcription and real time PCR	decrease viability of cells, expression of inflammatory genes, and expression of IL−6 and IL−8 genes	[73]
	human colonic epithelial cell CCD841CoN and small intestinal epithelial cell HIEC−6	PS	0.1, 0.5, 1, 5 μm	microspheres	500 μg/mL	24 h	CCK−8, FITC-AnnexinV-FITC/PI kit, JC−1 kit	low toxicity to oxidative stress level and mitochondrial membrane potential.	[74]
	small intestinal epithelium (SIE)	PS	25 and 1000 nm	microspheres			MTT, LDH	decreased viability and increased permeability	[75]
	Caco−2 and HT−29 cells	PE	19.2 μm		0.25 mg/mL, 0.5 mg/ mL, 0.75 mg/mL, 1.0 mg/mL	48-h	MTT, LDH, H2DCFDA, dihydroxy ethidium (DHE)	reduced cell viability and increased oxidative stress, mitochondrial superoxide production	[76]
	human intestinal epithelial cell line Caco−2 human cell lines Caco−2, HepG2 and HepaRG	PE, PP, PET and PVC	1–4 μm		100 μg/mL	24 h	MTT	Cytotoxic effects were seen when exposed to concentrations much higher than the actual human diet.	[77]
	Human intestinal epithelium in vitro	PS	20 and 40 nm			4–12 h	commercial live/dead cell assay, Vybrant Apoptosis Assay Kit	apoptosis - intestinal membrane damage	[78]
Kidney	Human renal cortical epithelial (HRCE) cell	PS	44 nm		40 μg/mL	72 or 168 h	MTT	Entry into HRCE cells via endocytosis	[79]
	In vitro Human embryonic kidney 293 (HEK293)	PS	3.54 ± 0.39 μm	spherical	3–300 ng/mL	24 h	DCFH-DA, JC−1, Human Inflammation Array 3 Kit (catalogue number: QAH-INF−3, BCA	Inhibition of antioxidant enzymes, cytotoxicity caused by ROS and Apoptosis and autophagy	[80]
	HK2	BPA,PE	1–4 μm		0.2–0.02 mg/mL		MTT Assay,” immunocytochemistry, western-blot, mRNA spectrophotometry analysis to evaluate the following biomarkers: MCP1, IL−1b, HSP90, Ahr, PGC−1a, NRF2, NOX−4	reduced the viability of HK2, upregulated MCP−1, IL1b, AhR and NOX−4, downregulating HSP90; NRF2 was downregulated by BPA and upregulated by MP	[81]
Respiratory tract	In vitro, Bronchus epithelial (BEAS−2B)	PS			1 mg/mL	24 h	MTS, ATP assay kit, RIPA, qRT-PCR	metabolic changes related to ER stress and autophagy	[82]
	Alveolar lung organoids cultured from epithelial cell progenitors, isolated both from murine lungs and human lung tissue.	Nylon or PE	11 × 30 μm and 15 × 53μm	microfibers		14 days	RNAseq,	Significant reduction of human respiratory organs	[83]
	Human lung epithelial cells (BEAS–2B)	PS	4.06 ± 0.44 μm		1–1000 *µ*g/cm2	24 and 48 h	Cytotoxicity assay, ELISA assay,DCFH-DA assays, Western blotassay, TEER	Cytotoxic - Oxidative stress and inflammatory response - damage of the epithelial layer	[84]
	human lung epithelial A549 cells	NP	100 nm		10, 20, 100, 200, 500 or 1000 μg/mL	24 h	CCK−8 assay, DCFH-DA, SOD assay kit, total CAT assay kit total, GSH-Px assay kit and MDA assay kit, (qPCR) analysis	oxidative stress and inflammatory	[85]
	Human lung adenocarcinoma cells (A549) (HT29)(PANC−1)	PS	100 nm		-	24 h	-	Enhanced cellular uptake of PS under fluidic shear stress	[86]
	human alveolar A549 cells	PS	1 and 10 μm		0–100 μg/mL	24–72 h	MTT	Decreased cell viability and Proliferation rate	[87]
	Human lung epithelial cells (Calu−3) and macrophages (THP−1cell lines)	PS	50 nm	Nanobeads			alamarBlue viability assay, γ-H2Ax foci	Increased DNA damages and Induced similar cytotoxic and genotoxic effects	[88]
	Caco−2, A549, and THP−1, HaCaT, U937, Jurkat	PTFE	6.0 or 31.7 μm		10–1000 μg/mL	24 h	CCK−8, BCA Protein Assay Kit	enhance inflammatory cytokine secretion And activating a signaling pathway	[89]
Hematopoietic	human leukocytic cell lines: Raji-B (B-lymphocytes), TK6 (lymphoblasts) and THP−1 (monocytes)	PS	50 nm	-	0–200 μg/mL	24 hand 48 h	Trypan Blue, DCFH-DA,	mild toxicity, ROS production and genotoxicity	[90]
	Caco−2, a human adenocarcinoma cell line with epithelial morphology	PS	50 nm and 0.5 μm		0.1–100 μg/mL	24 h	MTS assay	cellular uptake and intracellular accumulation	[91]
	Caco−2 cells	PET			0.1–100 μg/mL		MTS assay	Decreased cell viability and increased ROS production	[92]
	three human cell lines (A549, HEK293, and HeLa)	PS and PMMA	1.040 μm and 400 nm		10, 100, and 1000 μg/mL	24–72 h	Cytokine secretion	Changes in intracellular thiol content and cytokine secretion	[93]
	Caco−2 THP−1monocytic line	PS	4 µm and 10 µm			24–48 h	MTT, RT-PCR	Decrease in cell viability at very high doses.	[94]
	Human intestinal cell line HT−29	PS	3 and 10 µm		100–1600 particles/mL	24 h; 7, 14, 21, 28 and 48 days	Viability, Comet Assay, MTT	higher increase of ROS, reducing the rate of cell viability	[95]
	Human induced pluripotent stem cells (hiPSCs)	PS	100, 200, 500, 1000 nm	-	1–100 μg/mL	48 h	WST−8, RT-qPCR,	Cell viability decreased	[96]
Stem cells	Human serum albumin	NPs	100 nm		10, 25, 50, 75, 100 μg/mL	24 h	MTT,	genotoxic and cytotoxic	[97]
Immune	T98G and Hela cerebral & epithelial human cells	MNPs			10 ng/mL to 10 µg/mL	24–48 h	HCA, DHE	Oxidative stress	[98]
	Human Peripheralblood monocyticcells (PBMCs)U937 (humanmonocytic cell line)THP−1 (humanmonocytic cell line)	PS	(20–1000 nm)		10–2000 µg/mL		ATP, cytokines	cytotoxicity, IL−8 secretion, and oxidative burst, Induced IL−6 and IL−8 secretion	[99]
	Hs27 (Human fibroblasts)	PS	100 nm		5, 25, and 75 µg/mL	4, 24, and 48 h	MTS, (CBMN) Assay,	ROS production, genotoxic stress and DNA damage	[100]
	In vitro, Human Dermal Fibroblasts (HDFs), Human Peripheral Blood Mononuclear Cells (PBMCs), Red blood cells (RBCs) & the Human Mast Cell line (HMC−1)	PS	460 nm		500 μg /mL	24 h	MTT, ELISA kits	Liking of PS to RBCs caused hemolysis	[101]
	EA. hy926 cells	PS	3.9 ± 0.9 μm		4 × 10–6–40 μg/mL	24 h	trypan blue staining, DCFH-DA, BCA reagent kit	oxidative stress, apoptotic cytotoxicity	[102]
hepatic	human hepatocellular carcinoma (HepG2) cell line	PS	50 nm		10, 50 and 100 μg/mL		MTT, BCA protein assay kit,	Oxidative stress	[103]

**Fig. 4:**
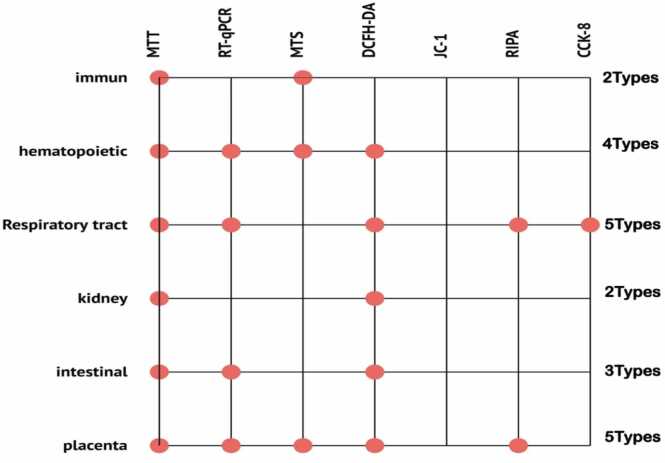
Number of cytotoxicity assays used in human cells based on Table 2.

**Fig. 5:**
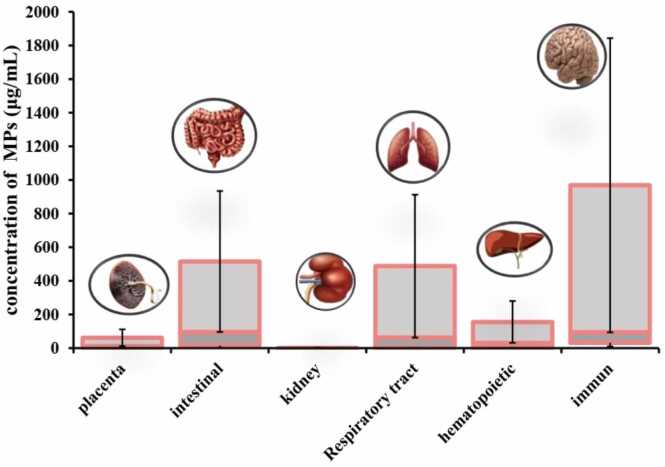
Comparison of exposure concentrations with human cell types, in the studies included in this review.

**Fig. 6:**
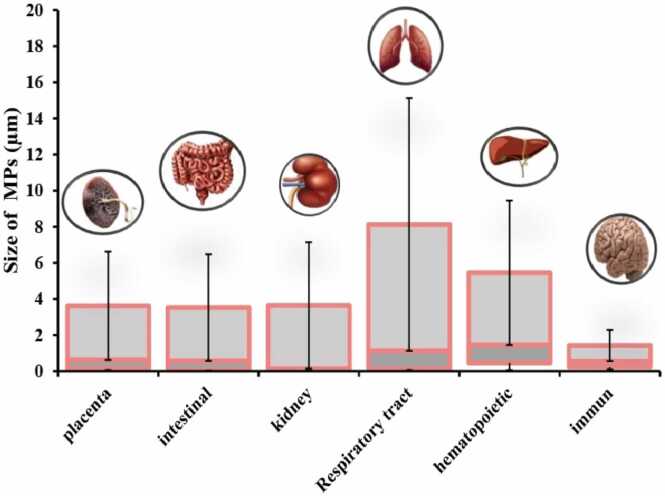
Comparison of exposure size with human cell types, in the studies included in this review.

**Fig. 7:**
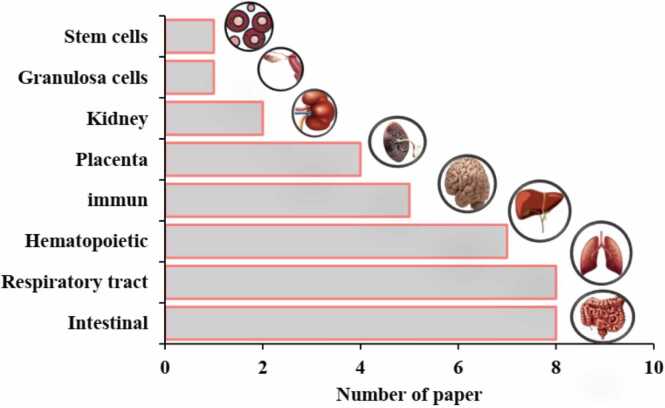
The number of paper studying the toxicity effects of MNPs in human cells.

**Fig. 8:**
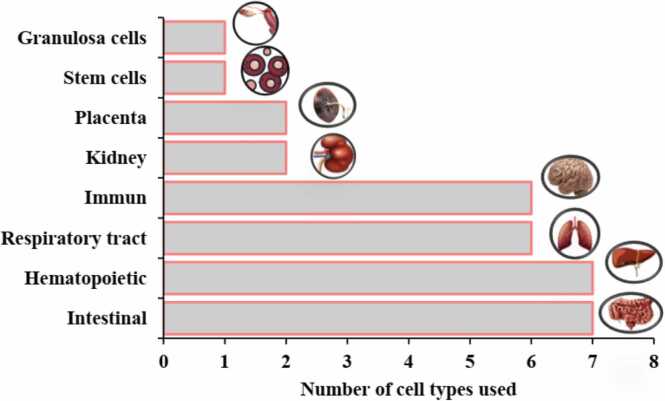
The number of cell types used, in the studies included in this review.

**Fig. 9:**
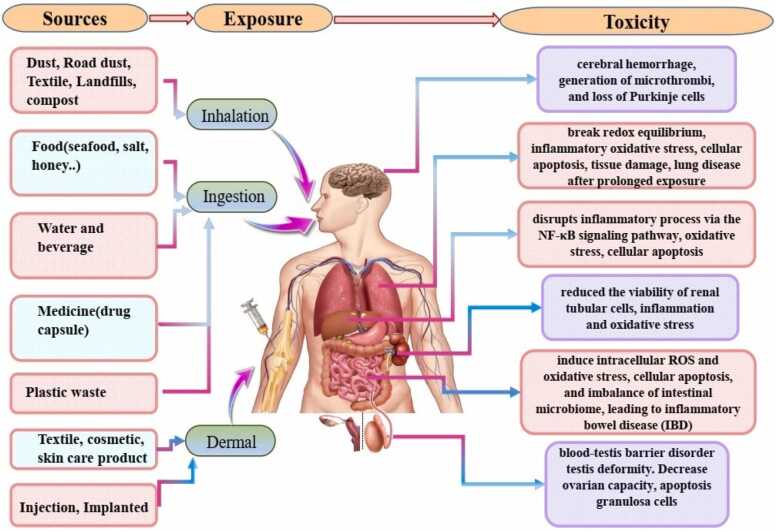
The schematic image first shows the different sources of MNPs and nanoplastics. Then it shows the three main routes by which humans are exposed to MNPs, i.e. inhalation, ingestion and dermal contact. This diagram also shows the effects of cytotoxicity due to the presence of MNPs in human body organs.

**Fig. 10:**
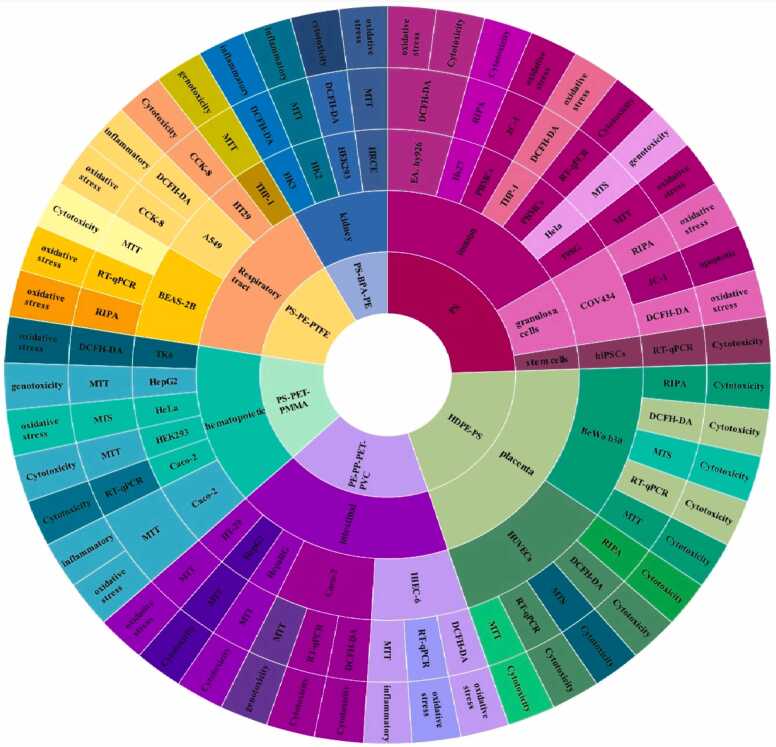
Sunburst diagram of cell models, MNPs polymers, toxicity tests and effects on cells reported by all studies in this review. Note: A549, adenocarcinoma human alveolar basal epithelial cells; BEAS-2B, human lung epithelial cells; BeWo b30, human placental choriocarcinoma cell line; Caco-2, human adenocarcinoma cell line; HeLa, cervical cancer cells; HepaRG, human hepatic cells; HepG2, Human Caucasian hepatocyte carcinoma cells; LDPE, low-density polyethylene; PBMCs, peripheral blood mononuclear cells; PE, polyethylene; PP, polypropylene; PS, polystyrene; T98G, human glioblastoma multiforme cell; HUVEC, Human umbilical vein endothelial cells; HIEC-6, isolated by thermolysin treatment of a human fetal small intestine; HEK293,Human Embryonic Kidney; HK-2 human kidney 2;THP-1, human leukemia monocytic cell line;HT-29,human colorectal adenocarcinoma cell; EAhy 926, hybridoma line derived from human endothelium; hiPSCs,Human-induced pluripotent stem cells; COV434,granulosa cell.

**Fig. 11:**
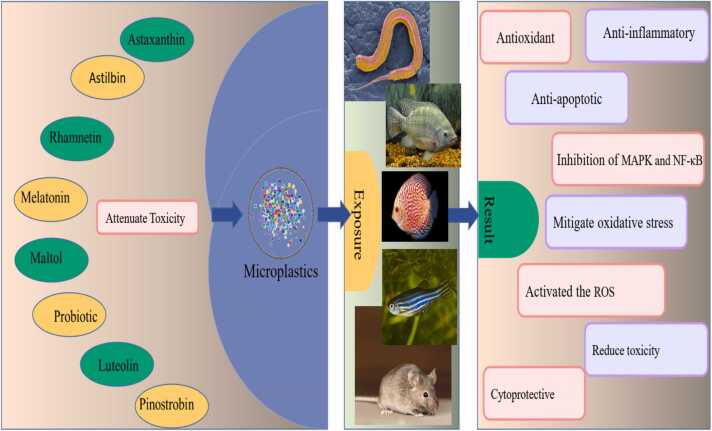
Summary of approaches used to counteract MNP toxicity, including environmental removal, dietary and antioxidant strategies, and biological mitigation methods.

The most frequently used cytotoxicity assays were MTT, DCFH-DA, and qRT-PCR (Fig. 3A). These tests were predominantly employed in lung and placental cells. The most frequently used tests were in lung and placenta cells, each employing approximately five types of tests, including MTT, DCFH-DA, qRT-PCR, RIPA, and CCK-8 (Fig. 4). In contrast, the immune system cells were tested the least, using only MTT and MTS (Fig. 4). The concentrations of MNPs used in these studies ranged from 0 to 2000 µg/µl (Fig. 5, Fig. 6, Fig. 7, Fig. 8, Fig. 9, Fig. 10, Fig. 11), with most exposure concentrations falling between 0 and 100 µg/µl. The exposure times in these studies varied, with 24 hours being the most common, followed by 48 hours (Fig. 3B).

The known research on MNPs and their potential toxicity is summarized in Table 2. As previously mentioned, humans can be exposed to MNPs through ingestion, inhalation, and dermal contact, which may lead to adverse health effects. Recent research has provided strong evidence that exposure to MNPs poses significant risks to human health (96, 97).

In summary, the studies reviewed indicate that respiratory and liver intestinal cells are the most frequently studied cell types in the context of MNP toxicity. The use of various cell models, such as Caco-2, THP-1, and A549, has provided valuable insights into the cytotoxic effects of MNPs. The findings highlight the importance of understanding the size and concentration of MNPs, as well as the duration of exposure, in assessing their potential health impacts. The diverse range of toxicity tests used in these studies underscores the complexity of MNP toxicity and the need for comprehensive research to develop effective mitigation strategies.[104], [105]. Therefore, in this section, we are going to focus on in vitro research of cytotoxic effects in human cells. Fig. 9 shows a summary of these toxicity effects on human cells.

#### 3.3.2 In vivo toxicity studies

Animal studies offer insight into systemic responses that cannot be fully captured in vitro. In rodent models, orally administered MNPs have been linked to alterations in gut microbiota composition, hepatic inflammation, and lipid metabolism disorders. Chronic exposure has been shown to result in accumulation in organs such as the liver, kidneys, and even brain, indicating potential for long-term damage. Notably, nanoplastics can translocate across the intestinal barrier, accumulate in the bloodstream, and distribute systemically.

Inhalation exposure to airborne microfibers in animal models has been linked to pulmonary inflammation and fibrosis-like pathology. Moreover, exposure to polystyrene nanoplastics has shown adverse effects on reproductive function and disruption of the hypothalamic–pituitary–gonadal axis in both zebrafish and rodents.

##### 3.3.2.1 Differential sensitivity based on particle characteristics

###### 3.3.2.1.1 Size

Nanoplastics (<100 nm) induce more profound cellular damage due to higher surface area and easier tissue penetration.

###### 3.3.2.1.2 Shape

Irregular fragments and fibers may elicit stronger inflammatory responses than spherical particles due to differences in cellular interaction.

###### 3.3.2.1.3 Polymer composition

Toxicity varies; for instance, PS and PVC often show greater biological reactivity than PE or PP due to leaching of monomers and additives.

###### 3.3.2.1.4 Additives

Endocrine-disrupting chemicals like phthalates and bisphenols, present as plasticizers, contribute to systemic toxicity, especially in endocrine and reproductive systems.

##### 3.3.2.2 Mechanisms of action

At the molecular level, MNPs activate stress-related pathways including MAPK, NF-κB, and Nrf2, leading to oxidative damage, inflammation, and cell cycle arrest. Mitochondrial dysfunction and DNA fragmentation have been noted in several models, suggesting a potential role in carcinogenesis.

While in vitro models offer mechanistic insights, in vivo systems are essential for evaluating whole-body and long-term effects. Integration of both approaches is critical for accurate risk assessment and setting safety standards.

### 3.4 Respiratory system

MNPs can be inhaled from different sources and are deposited in our lungs. As a respiratory organ and a potential target of air pollutants, the lung bears the major burden of MNPs in the air. Due to their characteristics, MNPs are easily inhaled into the lungs and cause toxicity, irritation, and inflammation. In a study, Dong et al. studied polystyrene with a size of about 4 μm with human lung epithelial cells (BEAS-2B). In this study, different concentrations between 1 and 1000 micrograms/mL were used, which showed that both concentration and size were two influencing factors on the level of cytotoxicity, oxidative stress, and inflammatory response[106]. In other studies, it has been reported that PS caused oxidative stress and endoplasmic reticulum stress, which led to autophagic cell death [82], [107]. After exposing human lung epithelial cells (Calu-3) and macrophages (THP-1 cell lines) to PS nanoparticles (50 nm), Padgett et al. showed that exposure to polystyrene nanoparticles leads to DNA damage, cytotoxic and genotoxic. Also, Xu et al. exposed human alveolar epithelial cell type II to PS nanoparticles (25 and 70 nm). The results showed that exposure to PS led to increased transcripts of NS-kB and inflammatory cytokines and the relationship between cell cycle and protein expression[108]. Studies have shown that the concentration of exposed MNPs, the size of exposed MNPs, and the duration of exposure all affect the level of toxicity created in lung cells [108]. PS-MPs lead to cytotoxicity and increased respiratory symptoms[86], [106], [109].

investigated how fluid shear stress influences cellular uptake as well as the mechanism of endocytosis of nanoparticles. In this study, human lung adenocarcinoma cells (A549) were exposed to 100 μg/mL of modified polystyrene nanoparticles (PSNs) with positive amino charge. The results showed that cancer cells absorbed a higher amount of PSNs in dynamic conditions. Moreover, in another study, [87] exposed human alveolar A549 cells to investigate the potential toxicological effects of MNPs on human cells. Cell proliferation was greatly reduced in both 1 and 10-micron sizes. No significant cytotoxicity was observed, and cell viability remained stable even at low exposure concentrations.

### 3.5 Gastrointestinal and urinary system

Many studies have been done regarding the toxicity effects of MNPs on the digestive and urinary systems, including the study conducted by[94], who exposed human intestinal cells to PS, which caused tissue and inflammatory effects inside the body. Other nanoparticles reduced cell viability and promoted apoptosis in all cell lines [110]. In another study, spherical polystyrene particles with three different sizes of 1, 4, and 10 µm were used to interact with human intestinal epithelial cell line Caco-2. which showed a negative effect on intestinal immune cells. [94]. Studies indicate that polystyrene nanoparticles adversely affect gene expression and increase inflammatory cytokine release (e.g., IL-6 and IL-8) in AGS gastric cells[73]. Reports indicate that exposure to smaller sizes of MNPs can cause inflammatory damage[111]. This pollutant can also cause toxicity in the urinary system. In a study conducted by[112], human renal cortical epithelial cells (HRCE) that were exposed to 44 nm polystyrene nanoparticles in the laboratory showed that the nanoparticles enter the HRCE cells through the process of endocytosis. In addition, another study showed that MPs damage the permeability of the intestinal barrier and reduce the population of bacteria present on the intestinal surface.[57], [58].

### 3.6 Blood and immune system

MNPs can act as foreign particles in the body, triggering immune responses and contributing to chronic inflammation. These particles have been found in human lung tissue, digestive fluids, and even the bloodstream, suggesting their potential to cause systemic immune activation. Long-term exposure may lead to increased levels of pro-inflammatory cytokines, exacerbating conditions such as asthma, inflammatory bowel disease (IBD), and autoimmune disorders. Moreover, MNPs may carry toxic chemical additives that further stimulate immune reactions, increasing the risk of allergic responses and inflammatory diseases.

Immune toxicity caused by MNPs is mediated through oxidative stress mechanisms involving the production of reactive oxygen species (ROS) and danger-associated molecular patterns (DAMPs) [113]. The produced ROS leads to disruption of the electrons pass through in a series of redox reactions, and The ROS-induced oxidative burst is largely attributed to the activation of NADPH oxidases (NOXs), leading to cellular stress and inflammatory signaling[113]. MNP particles (polyethylene) may completely replace lymph nodes when they are absorbed by macrophages [114]. The researchers noticed that when immune cells were exposed to acrylonitrile butadiene styrene (ABS) and PVC, increased the production of interleukin 6 (IL-6) and tumor necrosis factor-α (TNF-α) and induced inflammatory responses. The immunotoxic effects may be transient but require further validation through long-term studies[115]. In addition, exposure to small-sized particles produced large amounts of IL-6 products. Another study showed that when human serum albumin was exposed to NPs, changes were made in the protein structure and genotoxic and cytotoxic effects were seen. In a study conducted by[90], three different human leukocyte cell lines, Raji-B (B lymphocytes), TK6 (lymphoblasts), and THP-1 (monocytes) were exposed to PS at different concentrations and sizes of about 50 nm. The results showed that while monocytic THP-1 cells showed high uptake, Raji-B, and TK6 showed lower uptake of PSNPs, but no adverse effects were observed in monocytic THP-1. On the other hand, low toxicity, ROS production, and genotoxicity were seen in Raji-B and TK6. These findings highlight the importance of selecting appropriate cell lines for assessing MNP toxicity in immune cells[90].

### 3.7 Embryos and placental

The potential exposure of pregnant women to microplastics raises significant concerns regarding maternal and fetal health. However, the presence of microplastics in the human placenta has added to these concerns. Researchers found that microplastics in the placentas of pregnant women caused oxidative stress, cell death, and an inflammatory response. Also, the amount of MNPs in the placenta has an inverse relationship with the weight and height of the baby.[59], [60], [116]. Recent research on human embryonic stem cells and pluripotent stem cells (hiPSCs) by [117] has shown that carbonic anhydrase-IV (CA4) and ovarian cancer liver metastases (OCLM) are correlated with eye development. In a study, the interaction effect and autophagy of polystyrene nanoplastics (PS-NPs) (in sizes 100 and 500 nm) on human umbilical vein endothelial cells (HUVECs) were investigated. The results showed that exposure to 25 μg/mL of 500 nm MNPs for 48 hours significantly increased lactate dehydrogenase secretion from HUVECs[66]. In a similar study, the effect of polystyrene microplastics (PS-MPs) on human umbilical vein endothelial cells (HUVECs) was investigated. The results showed that 34 % decrease in viability HUVEC cells exposed to PS-MPs decreased dramatically. After that, autophagic cell death and necrosis increased. Also, absorption in cells was dependent on two factors, size and dose. [67]. Other studies investigated this toxicity effect in granulosa cells COV434 cells. The results showed that ovarian reserve was found to decrease significantly, whereas oxidative stress and apoptosis levels increased[70].

### 3.8 Nervous system

Emerging research indicates that microplastics can cross biological barriers, including the blood-brain barrier, raising concerns about their impact on neurological health. Once in the brain, MNPs may induce oxidative stress, neuroinflammation, and cellular damage, potentially contributing to neurodegenerative diseases such as Alzheimer’s and Parkinson’s. Studies on animal models have demonstrated that exposure to microplastics can impair cognitive function and alter neurotransmitter activity. While human studies are still limited, the detection of MNPs in brain tissue highlights the need for further investigation into their long-term effects on the nervous system.

In a study, two different cell lines, T98G and HeLa, were used, respectively, human brain and epithelial cells, and PS MPs (10 µm) and PS NPs. (40 and 250 nm) were exposed. The results showed that the particles are capable of inducing oxidative stress and cytotoxicity. These findings suggest that NPs may contribute to central nervous system pathologies through mechanisms involving oxidative stress and neuroinflammation. However, neurotoxic risk assessment requires in-depth investigation.[98].

## 4 Strategies for reducing microplastic toxicity and elimination

Given the widespread presence of microplastics (MNPs) in the environment and their potential health risks, developing effective strategies to minimize exposure and mitigate toxicity is crucial. This section explores current approaches to reducing MNP contamination in food and water, enhancing biological detoxification, and adopting lifestyle changes to limit human exposure.

### 4.1 Removal of microplastics from the environment and food

#### 4.1.1 Advanced filtration and water purification

One of the most effective ways to reduce MNP exposure is through filtration technologies designed to remove microplastics from drinking water and food sources. Advanced water treatment processes, such as membrane filtration (nanofiltration and reverse osmosis), have demonstrated high efficiency in trapping microplastic particles. Research has shown that activated carbon filters and ultrafiltration membranes can significantly reduce microplastic concentrations in tap water.

#### 4.1.2 Enzyme-based and bioremediation technologies

Recent advancements in biotechnology have introduced the use of plastic-degrading enzymes and microorganisms to break down microplastics. Certain bacteria, such as *Ideonella sakaiensis*, have been identified for their ability to degrade polyethylene terephthalate (PET). Additionally, enzymes like PETase and MHETase show potential in accelerating the breakdown of plastic polymers in natural environments, reducing their persistence in the food chain.

#### 4.1.3 Food processing and contamination prevention

Since microplastics are commonly found in seafood, salt, and processed foods, improvements in food processing methods are necessary to reduce contamination. Washing food items with filtered water, avoiding plastic packaging, and using glass or metal storage containers can help prevent additional microplastic contamination during food handling.

### 4.2 Biological strategies for reducing toxicity in the body

#### 4.2.1 Nutritional strategies for plastic detoxification

Minimizing the harmful effects of microplastics (MNPs) within the body requires biological strategies that enhance detoxification and reduce oxidative damage. Nutritional interventions play a critical role in mitigating MNP-induced toxicity by supporting digestive health, combating oxidative stress, and facilitating the elimination of these particles. High-fiber diets contribute to gut motility, helping to reduce the retention of ingested MNPs in the digestive tract and promoting their excretion. Additionally, bioactive compounds such as polyphenols, commonly found in green tea, berries, and turmeric, along with omega-3 fatty acids, have demonstrated the ability to counteract oxidative stress, a primary mechanism by which MNPs induce cellular toxicity.

Beyond dietary components, proper hydration and the intake of natural detoxifying agents may aid in the removal of MNPs from the body. Consuming adequate water supports renal filtration and toxin elimination, while certain foods, such as chlorella and spirulina, have shown potential in binding to harmful substances and facilitating their excretion. These dietary strategies, combined with a well-balanced intake of antioxidants and essential nutrients, may help mitigate the physiological effects of MNP exposure and improve overall resilience against plastic-induced toxicity. However, further research is necessary to fully understand the mechanisms behind these protective effects and optimize strategies for reducing MNP accumulation in human tissues.

While dietary strategies such as fiber intake, antioxidants, and hydration play a crucial role in mitigating MNP toxicity, emerging research highlights the potential of specific bioactive compounds in providing additional protection. Certain naturally occurring molecules, including melatonin, astaxanthin, probiotics, luteolin, pinostrobin, and astilbin, have demonstrated significant antioxidant, anti-inflammatory, and protective effects against MNP-induced cellular damage. These compounds work through various mechanisms, including the inhibition of oxidative stress, reduction of inflammation, and enhancement of cellular detoxification pathways. The following section explores the therapeutic potential of these bioactive compounds in alleviating the toxic effects of microplastics on human health.

##### 4.2.1.1 Melatonin

Melatonin is a neuroendocrine hormone secreted by the pineal gland, known for regulating circadian rhythms and other physiological functions [118]. It possesses anti-apoptotic, antioxidant, and anti-inflammatory properties[119]. In one study, male rats were exposed to MNPs once, and then they were exposed to melatonin and MNPs the second time. The results showed that MNPs caused Liver damage, cell necrosis, and inflammatory effects. However in the second exposure to melatonin in the group treated with melatonin, toxicity and liver damage were reduced. Also, in a similar study on zebrafish, their results showed that treatment of PS with melatonin shortened the length of cap motor neuron axons and decreased the expression of genes related to neural development. [120]. In another study, the protective effect of melatonin was measured in a plant. The results showed that melatonin activates the ROS inhibitor system, which means that ROS is less produced and oxidative stress is reduced. And it improves the metabolism of carbohydrates, so the plant grows more and increases tolerance to toxicity[121].

##### 4.2.1.2 Astaxanthin

Astaxanthin, a member of the xanthophyll family, has been extensively studied for its antioxidant, anti-apoptotic, and anti-inflammatory effects. It inhibits various molecular pathways, such as MAPK and NF-kB.[122]. One study found that astaxanthin reduced inflammatory responses induced by polystyrene microplastics (PS-MPs) in tilapia kidney cells by downregulating MAPK (p38MAPK and ERK1/2) and NF-kB signaling pathways.[123]. Another similar study conducted by Jun-Nan Huang showed that the oxidative stress induced in discus fish (red skin color) by MNPs was reduced by astaxanthin (ASX) and improved the antioxidant defense status[124].

##### 4.2.1.3 Probiotics

Probiotics, such as Bifidobacterium breve M-16V (B. breve M-16V), have shown promise in reducing MNP toxicity [125]. showed that B. Breve M-16V has anti-inflammatory and modulated PS-induced immunotoxicity. Also, [126]investigated the effects of probiotics on liver oxidative stress caused by polystyrene (PS)-MPs in Nile tilapia fish (Oreochromis niloticus). The results showed that probiotics reduced the oxidative stress caused by PS-MPs by restoring the activity of antioxidant enzymes and increasing the redox state.

##### 4.2.1.4 Luteolin

Luteolin has been investigated for its potential to reduce MNP toxicity in the reproductive system of male rats. Mohammad Omar Ijaz et al. showed that luteolin reduces all the disorders shown in the testes. PE-MP can cause testis damage in male rats, which is effectively reduced by LUT treatment[127].

##### 4.2.1.5 Pinostrobin

Pinostrobin has demonstrated significant potential in reducing oxidative stress and ROS levels, increasing sperm mobility, and mitigating testicular damage caused by PS-MPs. It is considered a promising therapeutic candidate for restoring testicular health affected by MNPs [128].

##### 4.2.1.6 Astilbin

Astilbin, known for its anti-inflammatory, anti-apoptotic, antioxidant, and androgenic properties, has been shown to protect against testicular damage caused by PS-MPs.

A summary of studies on agents that reduce MNP toxicity is provided in Table 3 and illustrated in Fig. 11. These studies highlight the potential of various compounds, such as melatonin, astaxanthin, probiotics, luteolin, pinostrobin, and astilbin, in mitigating the adverse effects of MNPs. Further research is needed to fully understand the mechanisms of action and to develop effective strategies for reducing MNP toxicity in humans [129].

**Table 3:** Approaches used to counteract the toxic effects of MNPs exposure.

Species	Chemicals and Strategies	Prevention Approaches and Effects	Ref
*Rat*	Melatonin	attenuated MNPs and ischemia-reperfusion toxicity.	[130]
*Zebra fish*	Melatonin	attenuated neurodevelopmental toxicity of PS by activating nrf2 - isl2a axis.	[120]
*tilapia head kidney cells*	Astaxanthin	inhibited the PS-MPs-induced stimulation of phosphorylation of the p38 MAPK, ERK1/2, and Iκκα/β and prevented PS-MPs-induced NF-κB nuclear accumulation	[123]
*discus fish*	Astaxanthin	cannot completely counter the oxidative stress caused by MNPs	[131]
*Rat*	Probiotic	restoration the gut microbiota balance. improve sperm quality and testicular inflammation caused by PS-MPs.	[132]
*Nile tilapia (Oreochromis niloticus)*	Probiotic	alleviated PS-MPs-induced oxidative stress by restoring antioxidant enzyme activities and reducing oxidized glutathione and enhancing the redox state	[126]
*Rat*	Luteolin	Increased sperm motility, viability. Reduction of sperm morphological abnormalities (head-tail and middle part). Increase of luteinizing hormone (LH), follicle stimulating hormone (FSH) and testosterone.	[127]
*Rat*	Astilbin	Its anti-inflammatory, anti-apoptotic, antioxidant and androgenic nature protected against testicular damage caused by PS-MPs.	[129]
*Rat*	Epigallocatechin−3gallate	optimized gut microbial composition, improved intestinal barrier function, reduced peripheral inflammation, and exerted anti-anxiety effects by inhibiting the hippocampal TLR4/MyD88/NF-κB signaling cascade.	[133]
*Rat*	Rhamnetin	anti-oxidant anti-apoptotic, anti-inflammatory as well as androgenic properties.	[134]
*Rat*	Maltol	promoted TFEB nuclear translocation through the AMPK/mTOR signaling pathway to restore lysosomal function and reduce autophagy dependent apoptosis.	[135]
*Rat*	Pinostrobin	escalated the activities of anti-oxidant enzymes and decreased MDA as well as ROS levels. restored the levels of inflammatory and apoptotic markers increased the expression of steroidogenic enzymes and hormone levels. improved all the histopathological alterations in the testicular tissues	[128]
*Rat and Caco2 cells*	Cyanidin−3-O-glucoside	Triggers autophagy by activating the Sirt1-Foxo1–1 signaling pathway to alleviate polystyrene-induced toxicity • The co-localization of polystyrene and lysosomes was observed, suggesting that PS is encapsulated and degraded • The co-localization of autophagy genes and PS was found, suggesting that autophagy is involved in the beneficial effects of C3G	[136]
*Rat*	Cyanidin−3-O-glucoside	remodels the gut microbiota and affects the gene abundance of bacterial functional pathways • Significantly increased levels of probiotics	[137]
*Rat*	Cyanidin−3-O-glucoside	regulates intestinal microbiome disturbance and regulates inflammatory function genes reducing bacterial functional genes associated with disease and inflammation	[138]
*C. elegans and Caco2 cells*	Cyanidin−3-O-glucoside	Recovery of polystyrene-induced ATP reduction, achieved by activating the AMPK/SIRT1/PGC−1α signaling pathway and by improving mitochondrial dysfunction and Increased fecal polystyrene efflux	[139]
*C. elegans*	Cyanidin−3-O-glucoside	ameliorate polystyrene-induced oxidative stress enhance the expression of DAF−16 pathway-related genes	[140]
*C. elegans*	Cyanidin−3-O-glucoside	Promotes intracellular GSH production by activating the PMK−1/SKN−1 pathway and also reduces the production of ROS and O2- induced by polystyrene	[141]

### 4.3 Reducing human exposure to microplastics

#### 4.3.1 Lifestyle modifications to limit MNP ingestion

To minimize exposure to microplastics through dietary sources, adopting practical lifestyle changes in food storage and preparation is essential. One effective strategy is replacing plastic food containers with alternatives such as glass, stainless steel, or ceramic. These materials do not degrade or leach microplastic particles into food, ensuring safer storage conditions. Additionally, reducing the consumption of processed and packaged foods can significantly lower microplastic intake, as studies indicate that prolonged contact with plastic packaging increases contamination levels. Another crucial step involves avoiding plastic utensils and kitchenware, particularly when heating food. Using metal or paper straws and opting for non-plastic kitchen tools can prevent the release of microplastics, which occurs when plastic materials are exposed to heat. Such simple yet effective modifications can substantially reduce the risk of microplastic ingestion.

#### 4.3.2 Minimizing inhalation of airborne microplastics

Reducing airborne exposure to microplastics requires proactive measures to improve indoor air quality and limit the release of synthetic fibers. One effective approach is the use of air purifiers equipped with HEPA filters, which can capture fine airborne particles, including microplastics, thereby minimizing indoor air pollution. Additionally, selecting natural fiber-based clothing can significantly reduce microfiber shedding during laundering and wear. Synthetic textiles such as polyester and acrylic shed microfibers during regular wear and washing, contributing to microplastic contamination in both air and water. Opting for natural fibers like cotton, wool, or hemp provides a more sustainable and safer alternative, reducing the release of microplastics into the environment while promoting healthier living conditions.

#### 4.3.3 Policy and environmental initiatives

Addressing microplastic pollution on a larger scale requires systemic changes through policy interventions and public engagement. Implementing and enforcing government regulations on plastic production, recycling, and waste management can play a crucial role in reducing environmental contamination. Stricter policies on plastic manufacturing, improved recycling infrastructure, and incentives for biodegradable alternatives can help curb the release of microplastics into ecosystems. In addition to regulatory efforts, raising public awareness is essential in driving behavioral change. Educational campaigns highlighting the dangers of single-use plastics and promoting sustainable alternatives can empower individuals to make environmentally conscious choices. Encouraging consumers to reduce plastic consumption, opt for reusable products, and support eco-friendly packaging can collectively contribute to mitigating microplastic pollution. By combining policy-driven action with increased societal awareness, significant progress can be made toward reducing microplastic exposure and its long-term health and environmental impacts.

## 5 Conclusion

Plastics are ubiquitously integrated into modern life, raising significant concerns regarding human exposure and the bioaccumulation of micro- and nanoplastics (MNPs). Most current studies on MNP toxicity have focused on environmental contexts, non-mammalian models, and in vitro mouse models. While it is documented that MNPs accumulate in human tissues and organs, relatively little research has been conducted on the damage they cause. Only a limited number of studies have examined MNP toxicity in human cells, and information regarding exposure dose, duration, and influencing variables remains scarce.

Many studies include a small number of samples, highlighting the need for more robust studies with larger and demographically diverse sample sizes to enhance generalizability.

Toxicity reduction approaches have also been neglected. Although the presence of MNPs in human matrices is concerning, strategies to reduce their toxicity are not yet well understood. The field of MNP diagnosis in humans is still in its infancy, necessitating further studies to obtain a comprehensive understanding. More in-depth research is required on the factors affecting MNP toxicity, the knowledge related to MNPs, and the possible risks.

This article focuses on studies that have identified MNPs in various biological samples from the human body and reviews research on the toxicity of MNPs in human cells. Additionally, it summarizes studies related to preventive strategies. The findings indicate that most research has been conducted on respiratory and liver intestinal cells, with common models being Caco-2, THP-1, and A549 cells. The majority of the tested cells were intestinal, respiratory, and hepatic in origin.

The size of the polystyrene particles ranged from as small as 0.025 µm in placental cells to as large as 10 µm in lung cells. Dominant toxicity tests in these studies included MTT, DCFH-DA, qRT-PCR, and MTS. Lung and placenta cells were subjected to the most diverse range of tests, including MTT, DCFH-DA, qRT-PCR, RIPA, and CCK-8, while immune system cells were tested the least, using only MTT and MTS.

The concentrations of MNPs used in these studies varied from 0 to 2000 µg/mL, with most exposure concentrations falling between 0 and 100 µg/µl. The most common exposure times were 24 hours, followed by 48 hours. Overall, this review provides valuable insights into the toxicity of MNPs and paves the way for future in-depth studies. Further research is essential to fully understand the health impacts of MNP exposure and to develop effective strategies to mitigate these risks.Measuring microplastics (MPs) and nanoplastics (NPs) in human tissues, such as blood, fluids, and lung tissues, faces significant limitations. One major analytical challenge is the ultra-low concentration of MNPs in human tissues, which often falls below the detection limits of conventional techniques. Biological matrices like blood and lung tissues contain complex mixtures of organic and inorganic substances that can interfere with detection, making it difficult to obtain clear results.

Contamination is another critical limitation. During sample collection and analysis, there is a high risk of contamination from environmental sources, leading to false positives. Even in controlled lab environments, contamination from plastic labware and airborne particles remains a significant concern.

Standardization issues further complicate the process. There is no universally accepted standard protocol for the collection, preparation, and analysis of human samples for MPs and NPs. This lack of standardization, combined with variability in methods used across different studies, makes it challenging to compare results and draw consistent conclusions.

Particle characterization is also problematic due to the size and shape variability of MPs and NPs, as well as the diversity of polymer types, which require different analytical approaches. Additionally, MPs and NPs can interact with biological molecules, such as proteins and lipids, altering their properties and making them harder to detect.

In summary, the main limitations in measuring MPs and NPs in human tissues include low detection sensitivity, interference from complex biological matrices, high contamination risks, lack of standard protocols, variability in methods, and difficulties in particle characterization. Despite these challenges, advancements in analytical techniques are gradually improving our ability to detect and quantify MPs and NPs, but continued research and standardization efforts are essential to better understand the health implications of these particles.

## 6 Research gaps

Despite the growing body of evidence regarding micro- and nanoplastics (MNPs), substantial gaps remain in our understanding of their health effects, exposure dynamics, and safe thresholds. Addressing these gaps is essential for guiding future research, policymaking, and risk mitigation strategies.

### 6.1 Lack of longitudinal and human-based studies

The majority of existing studies are either in vitro or short-term animal models. There is a critical lack of longitudinal human studies that assess chronic, low-dose exposure to MNPs and its potential role in the development of metabolic, reproductive, or neurodegenerative disorders. Long-term epidemiological data are urgently needed to correlate environmental exposure levels with clinical outcomes.

### 6.2 Understudied organs and systems

While studies have focused heavily on gastrointestinal, hepatic, and pulmonary effects, other organs such as the placenta, brain, endocrine glands, and reproductive system remain significantly under-investigated. Emerging evidence of MNPs in the placenta and cerebrospinal fluid indicates the need to explore trans-barrier transport mechanisms, fetal exposure, and neurodevelopmental toxicity.

### 6.3 Lack of standardized toxicity testing protocols

Current toxicological studies vary widely in terms of particle type, dose, duration, and model system used. This inconsistency hampers data comparability and the formulation of regulatory standards. There is an urgent need for standardized protocols for MNP toxicity testing, including guidelines for particle characterization (size, shape, surface charge), realistic exposure concentrations, and multi-system models that simulate human physiology more accurately.

## 7 Future research directions

Future research should prioritize:

• Development of advanced in vitro multi-organ systems (organ-on-chip) to simulate real-time exposure and systemic effects
• Use of human-derived biomonitoring data to refine risk models
• Exploration of synergistic toxicity between MNPs and other environmental contaminants (e.g., heavy metals, pesticides)
• Evaluation of bioaccumulation and biotransformation pathways of nanoplastics in the human body
• Assessment of social and occupational exposure patterns, especially among high-risk populations like food industry workers, agricultural laborers, and urban residents

## CRediT authorship contribution statement

**Jaafarzadeh Haghighi fard Neamatollah:** Writing – review & editing. **Jahedi Faezeh:** Writing – review & editing, Writing – original draft.

## Ethical approval

Not applicable.

## Consent for publication

All authors consented to participate in drafting and publishing this manuscript.

## Consent to participate

All authors consented to participate in drafting and publishing this manuscript.

## Funding

The authors acknowledge the support of grant No. 02s45 from Ahvaz Jundishapur University of Medical Sciences (10.13039/501100005001AJUMS). We would also like to thank the Student Research Committee of AJUMS for their assistance.

## Declaration of Competing Interest

The authors declare that they have no known competing financial interests or personal relationships that could have appeared to influence the work reported in this paper.

## Data Availability

Data will be made available on request.
